# Longitudinal Impact of the COVID-19 Pandemic on Older Adults' Wellbeing

**DOI:** 10.3389/fpsyt.2022.837533

**Published:** 2022-03-08

**Authors:** Javier López, Gema Pérez-Rojo, Cristina Noriega, Jose Angel Martínez-Huertas, Cristina Velasco

**Affiliations:** ^1^Department of Psychology and Pedagogy, School of Medicine, Universidad San Pablo-CEU, CEU Universities, Madrid, Spain; ^2^Department of Cognitive Psychology, Universidad Autónoma, Madrid, Spain

**Keywords:** longitudinal study, older adults, personal strengths, personal growth, purpose in life

## Abstract

**Background:**

The COVID-19 pandemic is a major stressful life event. This pandemic is causing significant changes in older adults' daily life affecting their physical and mental health. Psychological wellbeing is a protective variable when facing adverse circumstances, like the COVID-19 pandemic. This study analyzes the impact of COVID-19 on older adults' psychological wellbeing (personal growth and purpose in life) over time.

**Materials and Methods:**

One hundred ninety-two people over 60 years old participated in a longitudinal study. Data were collected in three time points: during the lockdown on March 2020, when the lockdown finished (4 months after baseline), and during the third wave (10 months after baseline). We used latent growth curve models to assess the linear longitudinal trajectories of psychological wellbeing.

**Results:**

Older adults did not show worse psychological wellbeing over time. Age has a positive impact on purpose in life. Furthermore, being a male, worrying about adverse effects of COVID-19, family functioning, resilience, personal growth, and acceptance associated with purpose in life.

**Discussion:**

These results suggest that despite the difficult circumstances experienced during the COVID-19 pandemic, older adults have used protective variables for their psychological wellbeing.

## Introduction

The COVID-19 pandemic has spread quickly in most countries and it has had incidence everywhere. Spain has been one of the most damaged countries during the first wave. The COVID-19 ease of transmission and the greater impact caused in the population group of older adults have the potential to affect older adult's psychological wellbeing (1). Life purpose (experimenting life meaning and direction) and personal growth (developing actions that improve talents and potential) are the core components of psychological wellbeing among older adults (2, 3).

A previous cross-sectional study found that the perceptions and psychological strategies that older adults use to cope with COVID-19 related difficulties (e.g., social isolation, becoming infected, losing a loved one) are more relevant than the nature of COVID-19. Specifically, showing better perceived health, resilience, family functioning, gratitude, and acceptance were associated with higher levels of personal growth and purpose in life (4).

The longitudinal studies analyzing the COVID-19 impact on older adults' wellbeing have shown mixed findings. Stressful events and losses impact people very differently (5). For example, in a study conducted in Sweden, life satisfaction showed a marginal but non-significant increase over time (6). In another study conducted in Finland, a significant decrease in quality of life from 2017–2018 to 2020 was found (i.e., before and during COVID-19 lockdown) (7). Also, the quality of life of older adults receiving home care services significantly decreased during the pandemic compared with the previous year (8). Another study conducted in Canada found that quality of life and wellbeing worsened over time in the period between before lockdown and 3 months after the first lockdown began and between the period before lockdown and the second lockdown. In contrast, they did not find any difference between the two lockdown periods (9).

In contrast, quality of life total scores significantly increased from April–May 2020 to July–August 2020 for older adults from the UK (10). In a study conducted in Chile, the resilience levels tended to increase from the pre-COVID period to the COVID outbreak, but there was no association between increased resilience and decreased depressive symptoms over time (11).

Moreover, the studies analyzing the pandemic effects on older adults are limited. And the research studying older adults' mental health during the pandemic has mainly focused on mental health problems (e.g., loneliness, anxiety, and depression). Only a few studies have focused on older adults' personal strengths or wellbeing. Furthermore, most studies considering mental health variables during the pandemic include the younger population who answered online surveys (4, 11, 12).

Given the potential impact that the COVID-19 pandemic may have on older adults' mental health, it is necessary to identify protective factors of their psychological wellbeing (8). For this, the development of longitudinal studies that evaluate the impact of the pandemic at different time points are needed (6, 10). The stress process model developed by Lazarus and Folkman proposes that primary stressors (such as COVID-19) can cause distress in the person. However, the impact of the stressor on the person's wellbeing depends on how they perceive the situation and the personal resources they put into practice to cope with it (13). Considering this model, we conducted a longitudinal study in which we analyzed the psychological wellbeing experienced by older adults during the COVID-19 pandemic and variables associated.

## Materials and Methods

Participants were older adults over 60 years old living in the community in Spain. We collected participants through the snowball sampling technique, social media (Twitter, WhatsApp, LinkedIn) and older adult's organizations. The first assessment (baseline wave 1) was a previous cross-sectional study of community-dwelling older adults from Spain assessed 3 weeks after the beginning of the lockdown restrictions (4). The second assessment (wave 2) was completed in July 2020 (a period without lockdown characterized by social distancing measures, some mandatory restrictions and a low rate of infections). The third assessment (wave 3) took place in January 2021 (during the third wave of the COVID-19 pandemic, which involved a high rate of infections and confirmed COVID-19 deaths and new restrictions were set). Participants who completed the survey in at least two time points were included in the study. In total, 64 participants were eliminated because they did not meet inclusion criteria: 47 were not living in Spain, and 17 were not living in the community. More specifically, 192 people aged 60 years or older completed the survey in T1, 167 in T2, and 126 in T3. In total, 192 participants responded the survey on T1 and T2. Most responded all the questionnaires on T1 and T2 (*n* = 167 participants). Nevertheless, some participants responded the survey on T2 but not all the questionnaires and they were excluded from the analyses (*n* = 25 participants). Moreover, 126 participants responded all the questionnaires on T1, T2, and T3.

Data were collected through a web-based survey. We included sociodemographic characteristics, self-perceived health, and aspects related to the COVID-19 pandemic. We also administered the following standardized questionnaires:

- The Family APGAR (14). The scale includes 5 items. They cover a person's family functioning (adaptability, partnership, growth, affection, and resolve). There are three response options ranging from 0 (hardly ever) to 2 (usually) and the sum score from 0 to 10. Higher scores indicate higher family functioning. We used the Spanish version (15), which showed good reliability in our sample (Cronbach's αT1 = 0.840; αT2 = 0.827; αT3 = 0.713).
- Brief Resilient Coping Scale (BRCS) (16). The scale includes 4 items. They cover a person's resilience. Response options range from 1 (nothing) to 5 (a lot) and the sum score from 4 to 20. Higher scores reflect higher levels of resilience. The Spanish version (17) showed adequate reliability in our sample (Cronbach's αT1 = 0.742; αT2 = 0.772; αT3 = 0.876).
- Gratitude subscale of the Values in Action Inventory of Strengths–Short Form (18). The subscale includes 5 items. They cover a person's gratitude. Response options range from 1 (very different to me) to 5 (very similar to me) and the sum score from 5 to 25, with higher scores indicating higher gratitude. The Spanish version (19) showed good reliability in our sample (Cronbach's αT1 = 0.868; αT2 = 0.900; αT3 = 0.924).
- The Acceptance and Action Questionnaire—II (AAQ-II) (20). The scale includes 7 items. They cover a person's experiential avoidance and psychological inflexibility. Response options range from 1 (not at all true) to 7 (completely true) and the sum score from 7 to 49. Higher scores show higher experiential avoidance and psychological inflexibility. The Spanish version (21) showed good reliability in our sample (Cronbach's αT1 = 0.899; αT2 = 0.896; αT3 = 0.899).
- Personal growth and Purpose in life Subscales of the Ryff's Psychological Wellbeing Scales (22). The subscales include 7 and 6 items, respectively. They cover a person's personal growth (how much they use their talents and potential) and purpose in life (how often they lives had meaning, and direction). Response options range from 1 (never) to 7 (always). Higher scores reflect higher personal growth and purpose in life. These Spanish version (23) showed good reliability for both personal growth (Cronbach's αT1 = 0.748; αT2 = 0.832; αT3 = 0.825) and purpose in life (Cronbach's αT1 = 0.872; αT2 = 0.842; αT3 = 0.894).

An informed consent form was attached to the survey, and each participant consented to participate after reading and agreeing with the informed consent information.

To calculate the linear longitudinal trajectories of life purpose and personal growth, we conducted different latent growth curve models. When personal growth and life purpose showed a statistically significant change, a full model with time-invariant and time-varying predictors was fitted to analyze the correlates of change. We used lavaan package (24) in R software to analyze the models fit. To manage missing data, we used Maximum Likelihood (ML) and Full Information Maximum Likelihood (FIML) estimators.

## Results

A total of 192 community-dwelling older adults from Spain completed at least two measures and met the inclusion criteria. Most participants were women (69.7%), were living with their spouse or partner (56.7%), and reported having good (47.8%) perceived health. At baseline, 40 people had a close relative or friend who had been hospitalized, 25 lost a loved one due to COVID-19, 23 suffered from COVID-19 symptomatology, and 2 had been hospitalized. Table 1 shows means, SDs, and percentages of all the variables in the three time points.

**Table 1:** Descriptive analysis along the three measurement occasions of the longitudinal study.

	**Mean**	***SD***
Age	68.22	5.85
	***N***	***%***
Gender						
Men	58	30.3
Women	134	69.7
Marital status						
Single	28	14.6
Married	109	56.7
Divorced	22	11.5
Widower or widow	33	17.2
	**T1**		**T2**		**T3**	
	**%**		**%**		**%**	
Perceived health			
Poor	4.9		4.8		5.6	
Fair	23.1		17.8		14.3	
Good	47.8		53.1		60.3	
Very good	24.2		24.3		19.8	
	**%**		**%**		**%**	
	**Yes**	**No**	**Yes**	**No**	**Yes**	**No**
COVID-19 consequences						
Symptomatology	12	88	6.6	93.4	11.9	88.1
Hospitalization	1.2	98.8	3.6	96.4	1.6	98.4
Loved one hospitalization	23.1	76.9	31.7	86.3	26.2	73.8
Loss of a loved one	14.3	85.7	23.4	76.6	21.4	78.6
	**Mean**	***SD***	**Mean**	***SD***	**Mean**	***SD***
Purpose in life	28.8	5.5	28.6	4.3	29.3	5.0
Personal growth	31.5	5.7	32.2	5.5	31.9	6.1
Experiential avoidance	19.5	7.1	19.5	7.0	18.1	6.7
Family functioning	13.8	1.9	13.8	1.7	13.9	1.4
Resilience	16.2	2.9	15.4	3.0	15.9	3.5
Gratitude	7.7	2.9	7.6	3.1	7.6	3.1
Worrying about adverse effects of COVID-19	1.4	0.8	1.5	0.8	1.5	0.8

*T1–T3 = measurement occasion. N = 192 (T1), 167 (T2), 126 (T3)*.

### Linear Longitudinal Trajectories

Life purpose showed good data fit in the latent growth curve model [$\chi_{\left(2\right)}^{2}$ = 1.791, *p* = 0.408, CFI = 1.009, TLI = 1.00, RMSEA = 0.001 [0.001–0.138], SRMR = 0.032]. Purpose in life did not show a statistically significant intercept due to variables and was standardized based on the baseline (b = 0.003, SE = 0.065, *z* = 0.040, *p* = 0.968) and a statistically significant increment over time (b = 0.072, SE = 0.034, *z* = 2.120, *p* = 0.034). The intercepts' variance was statistically significant (0.451, SE = 0.086, *z* = 5.249, *p* < 0.001), while the variance of the slopes was fixed to zero (the covariance between the intercept and the slope was not-statistically significant: 016, SE = 0.029, *z* = 0.531, *p* = 0.596). These results mean that there is a general longitudinal linear increase of purpose in life among the study, and those individuals present differences in their initial status but they have a similar longitudinal growth.

On the contrary, personal growth presented a not-statistically significant intercept due to variables and was standardized based on the first time point (b = 0.019, SE = 0.071, *z* = 0.273, *p* = 0.785) and did not increase over time significantly (b = 0.037, SE = 0.037, *z* = 0.975, *p* = 0.329). The intercepts' variance was statistically significant (0.711, SE = 0.124, *z* = 5.730, *p* < 0.001), but the variance of the slopes was not-statistically significant (0.058, SE = 0.057, *z* = 1.011, *p* = 0.312). The covariance between the intercept and the slope was not-statistically significant (−0.048, SE = 0.069, *z* = −0.700, *p* = 0.484). Thus, no longitudinal growth was found for personal growth along the study.

### Longitudinal Trajectories With Time-Invariant and Time-Varying Predictors

Since purpose in life was the only subscale of psychological wellbeing that showed a statistically significant increase over time, a full latent growth curve model with time-invariant and time-varying predictors was fitted. We observed less fit to the data in this model compared with the baseline model. However, the model residuals in purpose in life were adequate ($\chi_{\left(108\right)}^{2}$ = 190.778, *p* < 0.001, CFI = 0.874, TLI = 0.884, RMSEA = 0.063 [0.048–0.078], SRMR = 0.066). Table 2 shows the estimates of these latent growth curve models.

**Table 2:** Results from latent growth curve model with predictors for purpose in life.

	**Purpose in life**		
	**Estimate**	***SE***	***z*-value**
**Intercepts and slopes**			
Intercept (mean)	−0.019	0.041	−0.456
Slope (mean)	0.083	0.025	3.306^**^
Intercept (variance)	0.067	0.024	2.833^**^
Slope (variance)	–	–	–
Intercept and slope covariance	0.008	0.008	0.936
**Path estimates**			
*Time-invariant predictors*			
Gender (ref: women) → Intercept	−0.171	0.402	−0.426
Gender (ref: women) → Slope	3.577	1.211	2.953^**^
Age (years) → Intercept	0.761	0.388	1.961^t^
Age (years) → Slope	0.179	0.879	0.203
*Time-varying predictors*			
Personal growth	0.301	0.030	10.086^**^
Avoidance	−0.211	0.032	−6.544^**^
Family functioning	0.067	0.027	2.461^*^
Resilience	0.189	0.033	5.648^**^
Gratitude	−0.221	0.032	−6.949^**^
Worrying about adverse effects of COVID-19	0.069	0.026	2.625^**^

*N = 192*.

** *p < 0.01*.

* *p < 0.05*.

*t = p < 0.10. ML and FIML estimations. Given that the continuous predictors were standardized, their estimations can be understood as standardized estimates. Time-varying predictor parameters were fixed to be equal across measurement moments*.

When considering the time-invariant predictors of purpose in life, age showed a significant positive association with its intercept, that is, there was a positive association of age with purpose in life. Gender showed a significant positive relationship with the slope of purpose in life, being men tending to present a more important increase of purpose in life than women. Regarding the time-varying predictors, we observed different covariances between the change of purpose in life and the change of the predictors. Specifically, there was a positive covariance with family functioning, resilience, and worrying about adverse effects of the pandemic, and principally with personal growth. We also found a negative covariance with avoidance and gratitude.

## Discussion

Given the significant impact that the COVID-19 pandemic is generating on the population worldwide, the developed scientific literature is extensive. Most studies include cross-sectional data and focus on the psychological influences of the COVID-19 pandemic. We examined the impact of this pandemic on wellbeing with a longitudinal study developed in older adults from Spain. Our first aim was to determine the level personal growth and purpose in life throughout the COVID-19 pandemic. Contrary to the expected negative impact of the pandemic across different governmental restrictions (1), we did not find any negative effect of COVID-19 on wellbeing. In fact, older adults aged 60–95 perceived a linear increase of purpose in life.

At the same time, the majority showed stable personal growth levels. Previous literature supported that while personal growth tends to decline with age, some stressful situations, such as cancer status, were found to slow the decline in personal growth (2). We found that a potentially stressful situation, such as the COVID-19 pandemic, has no significant impact on the levels of personal growth among older adults. Personal growth was not affected negatively by the conditions of the pandemic.

Because of the highest mortality risk in older adults and the forced or voluntary social isolation to keep the social distance, older adults have been described in social media as the population group most affected by the COVID-19 pandemic (11). Also, WHO (1) predicted a great pandemic impact among older adults' mental health. Nevertheless, our study showed that media channels and health organizations could, perhaps, have maintained an ageism attitude. Older adults are more capable and have more strengths than many professionals have considered. Older adults increased their psychological wellbeing. Specifically, their personal growth remained stable while their purpose in life increased over time.

Cross-sectional studies found vulnerabilities in purpose in life. Age by itself has shown negative correlations with purpose in life. However, purpose in life changes depending on how older adults cope with life events, finding improvements associated with several psychological processes such as flexible self-perceptions and the use of adaptive coping strategies. Purpose in life has increased in our study because the pandemic offers them opportunities to develop resources to overcome future challenges. COVID-19 could have increased older adults' flexibility and coping strategies to achieve previously valued goals (2). Aging is a time of personal discovery. There are opportunities to learn new skills (25). Moreover, situations valued as less satisfying before COVID-19 can be perceived now as more satisfying (6).

Furthermore, men and participants who experienced lower avoidance, better family functioning, higher resilience levels, and better personal growth reported higher purpose in life. Surprisingly, lower gratitude and worrying more about adverse effects of COVID-19 were associated with a higher purpose in life.

Our results also support that the advantages related to gender observed in the beginning of the COVID-19 pandemic (7) are not attenuated through the pandemic. The effects persist over time. The pre-existing gender inequalities—socially and economically—have been amplified by the pandemic (26). Male older adults experience not only better quality of life in the early part of the COVID-19 pandemic in Sweden than in previous years, but also a better purpose in life of life from the start of the pandemic to the second wave in Spain.

Older adults are better able at regulating their emotions due to increasing their attention to positive emotions and prioritizing emotional goals as they become increasingly aware of limitations on their time and lifespan. Therefore, they experience more psychological wellbeing (25). In contrast, suppression, the inhibition of emotion, is a negative predictor of wellbeing (2). Given these findings, it is not surprising that older people showing more experiential avoidance (i.e., the resistance to contact with their unwanted thoughts, emotions, or body sensations) were related in our study with less purpose in life.

Regarding participants' social links with their close relatives, they did not change during the lockdown. The telephone was the most used way to contact their family (8). Family functioning could be related to psychological wellbeing in our longitudinal study since it is a variable related to social contacts and social support (27). Moreover, the absence of relational strain (i.e., relational harmony) predicted higher wellbeing (2). Hence, determining ways to improve family functioning will be important to enhance wellbeing during the pandemic.

A significant positive relationship between resilience and purpose in life was found. Previous literature support that when coping with life events and difficulties, many people can increase their sense of purpose and life meaning (28, 29). Resilience is a developmental process in which people, including older adults, achieve a successful adaptation to adversity and stressful events. The COVID-19 pandemic is a major stressful life event that confronts older adults' resilience and purpose in life.

In line with other studies (30), gratitude is related to purpose in life negatively because it is positively related to recognizing others while it did not show any association with recognizing oneself. Gratitude is a self-transcendent emotion that facilitates a prosocial orientation toward others. Thus, we hypothesized that gratitude would involve the goals of being close to and helping another person. Gratitude is others-oriented instead of self-oriented, while purpose in life is more self-oriented. Moreover, this negative relation could be because the present research did not distinguish between actual gratitude and remote gratitude. However, more studies are needed to clarify if and how the others' motivation goals related to gratitude mediate the decrease of purpose in life.

A second unexpected result is the positive relationship found between worrying about the adverse effects of COVID-19 and purpose in life. Coinciding with previous studies (31), life purpose is related to being able to find meaning in life experiences, including difficulties. People who show higher levels of life purpose evaluate negative events in a more adaptive and proactive way, perceiving and giving them explanations with meaning linked to their personal values. Likewise, people with higher levels of life purpose have more varied resources to face life's challenges, buffering the possible negative effect of stressors using strategies that help regulate emotions in a more adaptive manner. In this way, they would be better prepared to respond to emotional demands more effectively. Worrying about the adverse effects of COVID-19 has varied throughout the longitudinal study. This variation has generated a series of negative consequences in individuals that have caused changes in the purpose in life of the participants to better cope with these changes in worrying about adverse effects of COVID-19. Nevertheless, future research is needed to delve deep further into this result and to analyze how worrying about adverse effects of COVID-19 is associated with the increase of purpose in life.

Older adults' personal growth during COVID-19 pandemic is associated with purpose in life positively. These are the core components of psychological wellbeing and are connected variables (3). Previous studies focused on aspects of personal growth using autobiographical memories, narratives of major life goals, and stories of life transitions found that all such elements of personal growth have been positively associated with wellbeing (2).

These findings have some limitations. First, our research study is limited by its small sample size and may not represent the cultural, geographic, and socioeconomic or financial characteristics of Spain. Participants (Spanish natives or migrants from other countries; retired, employed, or unemployed older adults, etc.) had universal and guaranteed access to health services. Nevertheless, we were able to compare changes in older adults' wellbeing across the COVID-19 pandemic due to its longitudinal design. Second, this study only considered older adults living in the community, without severe cognitive impairment, and who volunteered to complete a web-based survey. Future studies are needed to cover the impact on people living in nursing homes and with cognitive impairment. Furthermore, older adults who do not use social media or information/communication technology devices may be more vulnerable to social isolation, particularly during the pandemic. Quality of life for this group may be lower compared with those who are more confident and readily use technology and communication device. Further research is needed to focus on older adults who do not use social media or information/communication technology devices.

Third, the paper did not measure the outcomes before the COVID-19 lockdown as the first time of data collection was in March 2020. Nevertheless, age itself has been associated with declines in purpose in life and personal growth. Fourth, purpose in life and personal growth are two of the six psychological wellbeing described in Ryff's model. However, we included these two variables since they are key dimensions in psychological functioning and have been described in the scientific literature as the main components of eudaimonia (3).

Nevertheless, this study suggests that older adults maintained their psychological wellbeing levels during the COVID-19 pandemic. These results also support that pandemic by itself may not be as relevant for older adults' wellbeing as their appraisals and personal resources they use to cope with COVID-related challenges. Some sociodemographics also influenced older adults' wellbeing. Therefore, being male and older, worrying about adverse effects of COVID-19, family functioning, resilience, gratitude, and acceptance showed significant associations with purpose in life.

A previous longitudinal study has shown that older adults did not evidence higher depression or anxiety than during the initial lockdown (32). Our research does not go along with the ageist viewpoint of the vulnerable and frail older adults often offered during this pandemic. Our results highlight that older adults have psychological and social resources that helped them to cope with adversity. Older adults have got over past stressful situations that could be empowering them to master adverse situations such as the COVID pandemic.

Among older people, having a good purpose in life is an important personal wellbeing goal. Older adults are a heterogeneous group. Since chronic traumatic events are highly prevalent stressors among older adults and still many of them show high levels of life purpose, the development of resources that improve psychological wellbeing is needed (e.g., resilience, acceptance, and family functioning). However, the high levels of life purpose reflected in this study should not underestimate other mental health's indicators. Furthermore, more studies are needed to delve into mental health components to mitigate the impact of the pandemic on older people. Considering that despite vaccination there are no indicators that the pandemic will end shortly, older adults' wellbeing has to continue being evaluated, especially if strict restriction measures are set again.

## Data Availability Statement

The raw data supporting the conclusions of this article will be made available by the authors, without undue reservation.

## Ethics Statement

The studies involving human participants were reviewed and approved by Ethics Committee of the University of San Pablo CEU (reference 436/20/26). The patients/participants provided their written informed consent to participate in this study.

## Author Contributions

JL: conception and design, data interpretation, drafting and writing the article, and revising it. GP-R: design, data collection, and writing parts of the article. CN and CV: data collection and writing parts of the article. JM-H: data analysis and revising for critical analysis. All authors contributed to the article and approved the submitted version.

## Funding

This work was supported by Universidad San Pablo-CEU, CEU Universities (CEU-Santander, Grant Number: MCOV20V3).

## Conflict of Interest

The authors declare that the research was conducted in the absence of any commercial or financial relationships that could be construed as a potential conflict of interest.

## Publisher's Note

All claims expressed in this article are solely those of the authors and do not necessarily represent those of their affiliated organizations, or those of the publisher, the editors and the reviewers. Any product that may be evaluated in this article, or claim that may be made by its manufacturer, is not guaranteed or endorsed by the publisher.
